# Humoral Predictors of Malignancy in IPMN: A Review of the Literature

**DOI:** 10.3390/ijms222312839

**Published:** 2021-11-27

**Authors:** Enrico C. Nista, Tommaso Schepis, Marcello Candelli, Lucia Giuli, Giulia Pignataro, Francesco Franceschi, Antonio Gasbarrini, Veronica Ojetti

**Affiliations:** 1 Department of Internal Medicine, Università Cattolica Sacro Cuore, Fondazione Policlinico Universitario A. Gemelli IRCCS, 00168 Rome, Italy; enricocelestino.nista@policlinicogemelli.it (E.C.N.); tommaso.schepis@gmail.com (T.S.); lucia.giuli92@gmail.com (L.G.); antonio.gasbarrini@policlinicogemelli.it (A.G.); 2 Department of Emergency Medicine, Fondazione Policlinico Universitario, Università Cattolica del Sacro Cuore, 00168 Rome, Italy; marcello.candelli@policlinicogemelli.it (M.C.); giulia.pignataro@policlinicogemelli.it (G.P.); francesco.franceschi@policlinicogemelli.it (F.F.)

**Keywords:** IPMN, biomarkers, pancreatic cancer

## Abstract

Pancreatic cystic lesions are increasingly detected in cross-sectional imaging. Intraductal papillary mucinous neoplasm (IPMN) is a mucin-producing subtype of the pancreatic cyst lesions arising from the pancreatic duct system. IPMN is a potential precursor of pancreatic cancer. The transformation of IPMN in pancreatic cancer is progressive and requires the occurrence of low-grade dysplasia, high-grade dysplasia, and ultimately invasive cancer. Jaundice, enhancing mural nodule >5 mm, main pancreatic duct diameter >10 mm, and positive cytology for high-grade dysplasia are considered high-risk stigmata of malignancy. While increased levels of carbohydrate antigen 19-9 (CA 19-9) (>37 U/mL), main pancreatic duct diameter 5–9.9 mm, cyst diameter >40 mm, enhancing mural nodules <5 mm, IPMN-induced acute pancreatitis, new onset of diabetes, cyst grow-rate >5 mm/year are considered worrisome features of malignancy. However, cross-sectional imaging is often inadequate in the prediction of high-grade dysplasia and invasive cancer. Several studies evaluated the role of humoral and intra-cystic biomarkers in the prediction of malignancy in IPMN. Carcinoembryonic antigen (CEA), CA 19-9, intra-cystic CEA, intra-cystic glucose, and cystic fluid cytology are widely used in clinical practice to distinguish between mucinous and non-mucinous cysts and to predict the presence of invasive cancer. Other biomarkers such as cystic fluid DNA sequencing, microRNA (mi-RNA), circulating microvesicles, and liquid biopsy are the new options for the mini-invasive diagnosis of degenerated IPMN. The aim of this study is to review the literature to assess the role of humoral and intracystic biomarkers in the prediction of advanced IPMN with high-grade dysplasia or invasive carcinoma.

## 1. Introduction

**Figure 1:**
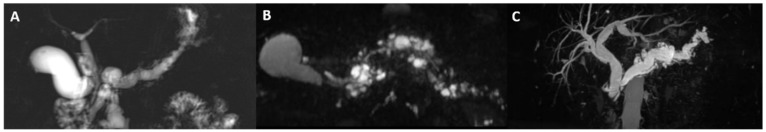
Magnetic resonance cholangiopancreatography (MRCP) showing: (**A**) Main duct Intraductal papillary mucinous neoplasm (IPMN), (**B**) Multifocal side-branch IPMN, (**C**) Mixed-Type IPMN.

Intraductal papillary mucinous neoplasm (IPMN) is a mucin-producing subtype of the pancreatic cyst lesions arising from the pancreatic duct system [1]. Depending on the involvement of the pancreatic duct system, we recognize three types of IPMN: main duct IPMN (MD-IPMN), branch duct IPMN (BD-IPMN), and mixed-type IPMN (MT-IPMN) when main duct, secondary branches, or both are involved respectively (Figure 1) [2]. Taking into account the histological structure and the mucin gene expression, IPMN can also be classified in gastric, intestinal, pancreaticobiliary, and oncocytic [3]. IPMN and mucinous cystic neoplasm (MCN) are considered precursors of pancreatic cancer and it is estimated that 8% of pancreatic malignancies arise from these lesions [4]. The transformation of IPMN in pancreatic cancer is a progressive oncologic process that begins as low-grade dysplasia, continues with high-grade dysplasia, and ends in invasive cancer. The precise mechanism of this process is not fully understood, yet but several genetic alterations have been identified as potential drivers of malignancy (e.g., KRAS, GNAS, TP53, and SMAD4 mutations) [5,6]. Unlike the other pancreatic cancer precursor (intraepithelial neoplasia-PanIN) which can be identified only with a histopathological examination, IPMN may be detected with cross-sectional imaging and can be classified as high or low risk for malignant transformation.

Jaundice, enhancing mural nodule >5 mm, main pancreatic duct diameter >10 mm, and positive cytology for high-grade dysplasia are considered high-risk stigmata of malignancy and are absolute indications for surgery. Increased levels of carbohydrate antigen 19-9 (CA 19-9) (>37 U/mL), main pancreatic duct diameter 5–9.9 mm, cyst diameter >40 mm, enhancing mural nodules <5 mm, IPMN-induced acute pancreatitis, new onset of diabetes, cyst grow-rate >5 mm/year are considered worrisome features of malignancy and relative indications for surgery [7]. Echoendoscopic Ultrasound (EUS) can be used to examine the morphologic characteristic of IPMNs to detect alarming features of malignancy with a sensitivity of 56–78%, and a specificity of 45–67% for the differential diagnosis between IPMN and other cystic lesions [8]. Differently from cross-sectional imaging, EUS allows the performance of Fine-Needle-Aspiration (FNA) for a biochemical (CEA, amylase, glucose, and mucin dosage) and cytological study of the cystic fluid [9]. Although cross-sectional imaging and EUS are considered the gold standard to detect, worrisome features of malignancy in IPMN, several biomarkers have been described as useful tools in daily practice. Carbohydrate Antigen 19-9 and Carcinoembryonic Antigen (CEA) are the most frequently used biomarkers during the follow-up [10]. Laboratory scores such as Neutrophil to lymphocyte ratio (NLR), Platelet to lymphocyte ratio (PLR), and C-reactive protein to albumin ratio (CAR) have been reported as potential predictors of malignancy in IPMN. Recently, cyst fluid DNA sequencing, microRNA (mi-RNA), circulating microvesicles, and liquid biopsy have been described as new frontiers for the mini-invasive diagnosis of degenerated IPMN. This study aims to review the literature to describe the evidence and current use of humoral biomarkers in the prediction of malignancy in IPMN.

## 2. Circulating Humoral Predictors of Malignancy

The risk of IPNM malignant transformation is not accurately predictable [Nasca2020]. For this reason, various clinical and radiological parameters have been considered to stratify the IPMN potential malignancy, thus identifying patients who will benefit from an early operation and those requiring a watchful waiting approach. Humoral predictors of malignancy can be crucial in the management of these lesions. Currently, an active area of research focuses on finding effective IPMN malignancy predictors, ranging from tumor markers as CA19-9 and CEA to serum inflammatory parameters such as NLR, PLR, and CAR.

### 2.1. Carbohydrate Antigen 19-9

CA 19-9 is a monosialogangloside expressed on the surface of cells first isolated in 1979 by Koprowski using a hybridoma made from a mouse’s spleen cells immunized with the SW116 human colorectal carcinoma cell line [11]. The CA 19-9 carbohydrate epitope, a sialylated lacto-N-fucopentanose I1 oligosaccharide, is related to the Lewis A blood group antigen [12,13] and it is not expressed in approximately 5–10% of the population because of the lack of 1, 4-fucosyl transferase enzyme, necessary for the sialyl Lewis antigen epitope production [14,15,16,17]. In physiological conditions, Ca 19-9 is produced by pancreatic and biliary ductal cells and secreted by gastric, colonic, endometrial, and salivary epithelia. Small quantities are present in serum as a high molecular weight glycoprotein complex. High levels of Ca 19-9 are found in the peripheral blood of patients with gastrointestinal cancers (e.g., adenocarcinomas of the stomach, gut, and pancreas) but also in benign diseases (peptic ulcers, pancreatitis, cirrhosis, cholangitis, and obstructive jaundice) [18,19]. Serum CA19-9 is the most consolidated tumor biomarker for pancreatic cancer, widely used for both adenocarcinoma diagnosis and prognosis [16,18,20,21]. In recent years, it has been investigated whether CA19-9 could be useful also for distinguishing invasive from benignant IPMN [22,23]. A large meta-analysis, which includes 15 studies and 1530 patients, reported a specificity for CA 19-9 of 89% and 88% and sensitivity of 40% and 52%, in malignant and invasive IPMN, respectively [24]. Evidence that elevated CA 19-9 values (>37 U/mL) are associated with both high-grade dysplasia and invasive cancer leads to its inclusion in several guidelines both as a “worrisome feature” and a relative criterion for resection [7,25]. Remain still unclear the correlation among CA 19-9 level with histological malignancy and survival. In this context, Cipriani et al. recently performed a single-institution large cohort study evaluating the utility of CA 19-9 in pathologically proven IPMN [26]. They demonstrate CA 19-9 as a predictor of IPMN malignancy and worse survival, even though with a reduced sensitivity that can limit the diagnostic value of the maker. More importantly, these results show that CA 19-9 is not closely associated with high-grade dysplasia but strongly correlated with more advanced stage tumors.

### 2.2. Carcinoembryonic Antigen

CEA is a 180 kDa cell-surface glycoprotein belonging to the immunoglobulin superfamily of cell adhesion molecules [27,28]. Gold and Freedman first discovered it from human colorectal cancer tissue and embryonic colon mucosa in 1965 [29]. A characteristic of the CEA family structure is the presence of several glycosylations on asparagine residues linked with multiantennary N-glycan chains that could present Lewis x and sialyl Lewis x structural motifs. They can play a role in the metastatic dissemination of colon carcinoma cells since they are biological ligands for L-selectin and E-selectin [30].

CEA is a cell-surface glycoprotein employed as a serum tumor biomarker, especially in colorectal cancer, as an independent predictor of overall survival, disease-free survival, and recurrence [31,32]. CEA is also used as a diagnostic value in pancreatic adenocarcinoma [33,34]. Furthermore, in last years, various studies give attention to its role to understand whether it could be useful in predicting malignant and invasive IPMN [22,23,26,35]. Fritz et al. demonstrated that CEA > 5 µg/L was present in 40% of patients with invasive IPMN and only in the 8% with a non-invasive IPMN, showing a sensitivity of 40% and a specificity of 92.4% [22]. A meta-analysis taking into account 15 studies published between 2001 and 2013 was reported by Wang et al., which demonstrated that serum CEA has low sensitivity (18%) and high specificity (93–95%) for malignant and invasive IPMN [35]. Kim et al. obtained similar results, showing a sensitivity of 6.1% and a specificity of 96.4% in predicting IPMN malignancy [23]. A recent study has shown the opposite conclusion: it found that increased values of serum CEA were not associated with a higher probability of malignancy in IPMN [26]. Therefore, the low sensitivity of CEA makes it unsuitable to be used as a screening method, especially in high-risk patients. However, considering its high specificity, this marker can be valuable in rule-in IPNM malignancy [36].

### 2.3. Neutrophil to Lymphocyte Ratio

The NLR is an inflammatory marker derived from the total neutrophils count divided by the total lymphocytes count. Its preoperative value, if elevated, correlates with poor prognosis in various solid malignancies [37,38]. In the IPMN context, Arima et al. found a higher NLR value in patients with IPMN invasive carcinoma compared to non-invasive IPMN and healthy volunteers [39]. In their study, an NLR > 2.074 presented a sensitivity of 73.1% and a specificity of 58% in predicting potentially malignant IPMN. A combined criterion, which includes this cut-off NLR value with other factors such as international consensus guidelines and CA 19-9 > 37, showed a high positive predictive value of 78% and high specificity of 96% [39]. In a retrospective study taking into account 272 patients with pathologically documented IPMN, Gemenetzis et al. demonstrated that a value NLR > 4 was an independent predictive marker for the presence of IPMN-associated invasive carcinoma [40]. However, the NLR sensitivity was not able to discriminate between the different degrees of dysplasia. Hata et al. also described the ability of NRL in predicting high-grade dysplasia/invasive-IPMN [41]. To combine NRL with image findings, CEA, and CA 19-9, increases the predictive power with a sensitivity of 58.8% and a specificity of 76.8%. This study also observes that high NLR values correlate with tumor aggressiveness and poor outcomes in IPMN invasive associated carcinoma [30]. Conversely, Onho et al. found no significant difference in prognosis between high NLR and low NLR [42]. McIntryre et al. confirmed that NLR was unable to discriminate high-grade dysplasia from low-grade dysplasia [43]. However, the NLR remains an easy to perform cost-effective diagnostic marker for predicting IPMN associated with invasive carcinoma, especially when combined with other factors. It is worth noting that the optimal cut-off value is still an open point.

### 2.4. Platelet to Lymphocyte Ratio 

PLR derives from the ratio between the total counts of platelets and lymphocytes. Similar to the NLR, it is an inflammatory marker with a pivotal prognostic role in various solid tumor cancers [44]. In a retrospective study involving 120 patients who underwent surgery for a pathologically proven mucin-producing pancreatic cystic neoplasm (MpPCN), PLR > 208.1 was an independent predictor of invasive carcinoma in MpPCN [45]. In patients categorized as “High risk” in the Fukuoka Consensus Guideline, high PLR was associated with 83.3% invasive carcinoma versus 42.5%, showing that the use of PLR could improve the positive predictive value of these guidelines in detecting invasive neoplasms [25,45]. On the other hand, the retrospective study by Gemenetzis et al. suggests that PLR is not a predictive marker for high-grade dysplasia or IPMN-associated invasive carcinoma [40]. Further investigations are hence necessary to fully assess its role.

### 2.5. C-Reactive Protein to Albumin Ratio

Host-related inflammatory biomarkers such as the NLR, PLR, among others, have been recognized as prognostic factors in pancreatic adenocarcinoma [46,47]. The significance of these markers in IPMN diagnosis is an intriguing aspect currently under investigation [40,41,48,49]. The role of the ratio between the CAR has been less investigated. Very recently, Serafini et al. conducted a retrospective analysis on 83 patients who underwent pancreatic resection for IPMNs [50]. Their results suggest that CAR > 0.083 was a statistically significant predictor of invasive carcinoma or high-grade dysplasia in IPMNs with a sensitivity of 52% and a specificity of 93%. Although CAR has low sensitivity in detecting malignancy, its specificity overcomes the ICG criteria for detecting malignant IPMN. Moreover, patients with low CAR value presents better overall survival when compared to those with CAR > 0.083 [50]. These findings indicate that CAR can be an easy-to-obtain indicator in IPMN clinical treatment. Although international multicentric clinical studies are needed to confirm its relevance, CAR may be promising when integrated with imaging analysis and consolidated tumor markers.

### 2.6. Cyst Fluid Sample 

When the cross-sectional imaging is inconclusive or documents worrisome features, EUS evaluation is frequently required. The EUS morphological assessment alone is generally inadequate to differentiate mucinous from serous cysts [51]. Thus, an FNA is performed to analyze a sample of the cyst fluid and increase the diagnostic accuracy of EUS. Classically, the cyst fluid is tested for CEA, amylase, and cytological analysis [52]. Recently glucose, mucin, and DNA sequencing in the cyst fluid are under evaluation for differential diagnosis of mucinous neoplasms.

### 2.7. Carcinoembryonic Antigen

Lewandrowski KB et al., firstly reported the dosage of tumor markers in pancreatic cyst fluid in 26 patients [53]. They documented high levels of CEA in all mucinous lesions and low levels in serous cysts and pseudocysts. Brugge W.R. et al. performed a multicenter retrospective study collecting the tumor markers dosage in cyst samples of patients undergoing surgery for final histological examination [8]. They demonstrated that cyst fluid CEA higher than 192 ng/mL presented a diagnostic accuracy of 88% in differentiating mucinous from a non-mucinous cyst. Laurens A. et al. reported that CEA levels lower than 5 ng/mL were predictive of a serous cyst with a sensitivity of 50% and a specificity of 95% [54]. The same authors documented that raising the threshold of cyst CEA to 800 ng/mL the diagnosis of mucinous cyst presented a higher specificity (98%) but a significantly lower sensitivity (48%). The threshold of 192 ng/mL is used in daily practice as a binary cut-off with a sensitivity of 52–78% and specificity of 63–91% in distinguishing between mucinous and non-mucinous cysts. The cyst CEA levels are not accurate for the differential diagnosis between IPMN and mucinous cystadenoma [55]. Moreover, cystic CEA is not adequate in predicting the presence of dysplasia or invasive carcinoma [56].

### 2.8. Cytological Analysis 

The specimens obtained with EUS-FNA can be used to perform a cytological analysis. Positive cytology for high-grade dysplasia or malignancy is considered an absolute indication for surgery [7]. According to the Papanicolaou Society of Cytopathology (PSC), the pancreatobiliary cytology can be classified into six diagnostic categories: (1) Non-diagnostic (a specimen that provides no diagnostic information about the solid or cystic lesion); (2) Negative for malignancy (a specimen that contains adequate cellular or extracellular tissue without consistent evidence of malignancy); (3) Atypical (a specimen that contains cells with cytoplasmic, nuclear, or architectural features that are not consistent with physiological or reactive changes); (4-A) Neoplastic (a specimen consistent with a benign lesion), (4-B) Neoplastic (a neoplasm that is pre-malignant or low-grade malignant); (5) Suspicious for malignancy (a specimen that presents features of malignancy, but the findings are qualitatively or quantitatively insufficient for a conclusive diagnosis); (6) Positive (a specimen that contains malignant cytological features) [57,58]. Thornton G. D. et al. performed a metanalysis of 18 studies with 1438 patients affected by IPMN that underwent EUS-FNA. The pooled sensitivity and specificity of cytological analysis in the differential diagnosis between mucinous and non-mucinous cysts were 54% and 93%, respectively [9]. Several cytological findings can help to distinguish the different pancreatic cists: for example, the presence of macrophages, histiocytes, and neutrophils is suggestive of pseudocyst; the presence of mucin is suggestive of mucinous neoplasm; the presence of glycogen-rich cuboidal cells indicate a serous cystic neoplasm [59]. Moreover, the cytological analysis can detect malignancy within the mucinous cists with a reported high specificity (83–99%) but a low sensitivity (25–88%) [59].

### 2.9. Glucose 

Recently, the dosage of glucose in the cystic fluid has been reported as a useful tool in the differential diagnosis between mucinous and non-mucinous cysts. Park G. W. et al., performed a metabolomic analysis on pancreatic cyst fluid samples and reported that the metabolomic abundance of glucose was lower in mucinous cysts [60]. Faias S. et al. reported similar results on cyst fluid samples collected with EUS-FNA. In this study the median glucose levels were 19 mg/dL (IQR 19-19) in mucinous and 105 mg/dL (IQR 96–127) in non-mucinous cysts (*p* < 0.0001) [61]. Glucose levels < 50 mg/dL had a sensitivity of 89% and a specificity of 86% in identifying mucinous cysts. Car R. A. et al. compared glucose and CEA levels in the cystic fluid of 153 patients with histologically confirmed diagnoses [62]. The median glucose level was 19 mg/dL in mucinous and 96 mg/dL in non-mucinous cysts. With the threshold of 50 mg/dL, glucose levels had a sensitivity of 92%, a specificity of 87%, and a diagnostic accuracy of 90% in the identification of mucinous cysts.

Although glucose seems comparable, if not superior to CEA in differentiating between mucinous and non-mucinous cysts, it is not adequate to detect degenerate IPMN with dysplasia or invasive carcinoma.

### 2.10. Mucin 

Mucins (MUCs) are highly glycosylated glycoproteins expressed in epithelial cells [52]. MUC can be detected by specific stains or by utilizing gene expression. MUCs research can be performed on cystic fluid sent for cytological and chemical analysis with no need for other specimens. MUC can differentiate the mucinous from the non-mucinous cyst with a reported sensitivity and specificity of 80% and 40%, respectively [63]. MUC 1 is not expressed in normal pancreatic tissue and is considered a marker of malignancy and invasiveness [64]. Nissim S. et al. in a metanalysis of 39 studies, reported that MUC 1 was detectable in 8.6% of not-degenerated IPMN and 35.8% of cancerized IPMN; MUC 2 was detectable in 51.7% of not-degenerated IPMN and 68.9% of cancerized IPMN; MUC 5A showed the weakest association with malignant progression of IPMN and was expressed in 84.7% of not-degenerated IPMN [65]. Moreover, MUC 4 was implicated in IPMN development with increased expression in degenerated IPMN [66]. The research of mucins in cystic fluid on one side can be useful to the differential diagnosis between mucinous and non-mucinous cysts, but, more interestingly, on the other one, has the potential to detect malignant and degenerated IPMN.

### 2.11. Amylase 

Cyst levels of amylase can be used as an indicator of communication with the pancreatic duct system. Amylase levels are generally high in pseudocysts but can also be detected in other cystic pancreatic lesions such as IPMN [67]. Waaij LA et al. in a review of 12 studies reported that amylase cystic levels lower than 250 U/L virtually excluded pseudocysts and can be found in mucinous and serous cysts [54]. IPMN presents, by definition, a direct connection with the pancreatic duct system while mucinous cystadenomas lack this connection; however, amylase can be detected in both cases and is not useful for their differentiation. In clinical practice, cystic amylase levels < 250 U/L can be used to exclude pseudocysts with a sensitivity of 44% and a specificity of 98% but it cannot be used either to differentiate between other non-mucinous and mucinous cysts or to detect degeneration or malignancy [7].

### 2.12. Other Intra-Cystic Markers 

CA 19-9 is a tumor marker widely utilized for the management of pancreatic and biliary malignancies. As said before, CA 19-9 is generally dosed in a peripheral blood sample with a threshold of 37 UI/mL. Moreover, CA 19-9 can be dosed in cystic fluid samples. Stigliano S. et al. performed a metanalysis of seven studies involving 939 patients to assess the role of intra-cystic CA 19-9 in the differential diagnosis of pancreatic cysts [68]. The reported sensitivity and specificity of CA 19-9 in distinguishing between mucinous and non-mucinous cysts were respectively 68% and 68% [68]. The high heterogeneity among the studies, the small population sizes, and the absence of a standardized cut-off are the major complaints of the studies available in the current literature thus not supporting the use of intra-cystic CA 19.9 in daily practice.

The dosage in cystic fluid samples of other markers such as CA 72-4, CA 125, and CA 15-3 has been evaluated in a few studies, however, their clinical role is still not defined, and more prospective studies are needed [8]. Ajay V. M. et al. evaluated the expression of cytokines in cystic fluid samples hypnotizing the role of immunogenic and pro-inflammatory microenvironment in the development of dysplasia and invasive carcinoma [69]. They reported that high-risk IPMN were associated with elevated levels of cytokines reflecting a Th1 and Th2 immunologic response. Specifically, they found that Interleukin-1b (IL-1b) levels in cystic fluid samples were higher in patients with high-grade dysplasia and invasive cancer compared with those with low-grade or moderate dysplasia. In the multivariate analysis, high levels of IL-1b were predictive of high-risk cysts. They concluded that IL-1b might represent a useful tool in the clinical setting to choose whether to perform surgery.

## 3. New Perspectives 

### 3.1. Cyst Fluid DNA Sequencing 

The progression from normal pancreatic cells to pancreatic cancer is a complex mechanism involving the accumulation of genetic variations such as gene mutations, gene down-regulation or up-regulation, and chromosomal aberrations [70]. As such, the transformation of IPMN into pancreatic cancer depends mainly on molecular mutations of proto-oncogenes or oncogenes leading to aberrant cell growth [71]. Therefore, the cyst fluid DNA analysis has been investigated as a tool in distinguishing between mucinous and non-mucinous cysts, and, more interestingly, in the early detection of malignancy. Khalid A. et al. performed a prospective multicenter study to evaluate the utility of DNA analysis of pancreatic cyst fluid to diagnose mucinous cists and degenerated lesions in 113 patients [72]. The reported elements of DNA analysis associated with malignancy were a high amount of pancreatic cyst fluid DNA, high-amplitude mutations, and high amplitude KRAS mutation. Cyst fluid KRAS mutation was predictive of mucinous cysts with high specificity (96%) but low sensitivity (45%).

Other studies reported the role of GNAS mutations in the progression towards pancreatic cancer. Among the several pancreatic lesions, the GNAS mutation at codon 201 is observed exclusively in IPMNs and is present more frequently in the intestinal subtype [73]. Kadayifci A. et al. evaluated the role of molecular analysis of pancreatic cystic fluid collected from 197 patients. They documented that by adding GNAS mutation to KRAS and CEA the diagnostic accuracy of IPMN was significantly increased (86.2%). Moreover, GNAS mutation can be used to distinguish carcinomas derived from IPMNs and concomitant pancreatic adenocarcinoma [74]. McCarty R. T. et al. performed a metanalysis of six studies involving 785 pancreatic lesions and documented that the combination of KRAS and GNAS in the molecular analysis of cystic fluid had a sensitivity, specificity, and diagnostic accuracy of 94%, 91%, and 97% in the diagnosis of IPMN and mucinous cysts [75].

RNF43 is another protein exerting a tumor suppressor activity; its mutations have been described in IPMN [76]. Chang X.Y. et al. performed a mutational analysis of 61 IPMN specimens and reported that RNF43 mutations were present only in high-grade dysplasia or invasive lesions [77]. They also showed that RNF43 was always associated with GNAS mutations and with a worse prognosis. However, RNF43 does not seem to be involved in the progression to invasive carcinoma but it plays a role in the transition from low to high dysplasia [78].

Other mutations such as TP53, SMAD4, and CDKN2A have been described in pancreatic adenocarcinoma and carcinoma derived from IPMN, however, their dosage in cystic fluid and their use in clinical practice have not been widely evaluated yet [79].

### 3.2. MicroRNA (mi-RNA) and Telomeres 

MicroRNA (miRNA) are small non-coding RNA that regulate gene expression at the post-transcriptional level. Different miRNA expression profiles are present at different stages of pancreatic malignancy [80]. Several studies documented that miRNA can have both oncogenic and onco-suppressor functions and that dysregulation of miRNA is associated with tumorigenesis and cancer progression, [81]. Wang J. et al. performed a Next-Generation Sequencing (NGS) study of miRNAs in the cyst fluids of pancreatic cystic lesions [35]. They stratified different grades of pancreatic cysts (low-grade, high-grade, and invasive carcinoma) and found that 13 miRNAs were increased, and two miRNAs were reduced in the cystic fluid of invasive carcinoma. Utomo W.K. et al. evaluated the accuracy of a nine-miRNA panel in distinguishing between high-risk and low-risk pancreatic cysts in 62 patients [82]. The reported sensitivity was 10.0%, the specificity 100.0%, the positive predicted value 100.0%, the negative predicted value 85.2%, and the overall diagnostic accuracy was 85.5%. Similarly, Shirakami Y. et al. described a panel of six miRNA enriched in IPMN with invasive carcinoma when compared with benign IPMN [83].

Telomeres are repeated sequences located at the end of all chromosomes, telomeres prevent the fusion of chromosomal ends, and telomeres shortening is a crucial mechanism to allow cellular apoptosis and to prevent cellular overgrowth [84]. Telomeres are enzymes, generally not expressed in normal cells but present in neoplastic cells, which maintain the telomeres length representing an important mechanism of neoplastic cells’ immortalization. Hata T. et al. evaluated the role of pancreatic cystic fluid telomere fusion in predicting the risk for high-grade dysplasia and invasive carcinoma in patients with IPMNs [85]. They documented that telomere fusion was more frequent in IPMNs with HGD (26.9%) and IPMNs with invasive cancer (42.9%) than IPMN with intermediate- or low-grade dysplasia (15.4% and 0% respectively). At the multivariate analysis, cyst fluid telomere fusion was an independent predictor of high-grade dysplasia and invasive carcinoma. Similarly, the same researchers, in another study reported that elevated cyst fluid telomerase activity has a diagnostic accuracy for invasive cancer and high-grade dysplasia of 88.1% [86].

### 3.3. Circulating Microvesicles 

Extracellular vesicles and particles (EVPs) from bodily fluids, plasma, and tissue explants, have been recognized as ideal diagnostic tools for multiple human cancers, serving as reliable biomarkers for early-stage cancer detection [87]. Specifically, EVPs identify cancer-specific proteins in tissues; a machine-learning analysis of EVP plasma cargo enables distinguishing tumors from normal tissues and among various cancer types [70]. In searching for a noninvasive stratification method for detecting high-risk IPMN, a recent relevant study focused on extracellular vesicle (EV) analysis and successfully predicted IPMN with invasive carcinoma [88]. Yang et al., in a study that enrolled 133 patients, divided into a discovery cohort (healthy controls and patients with HG- or LG- IPMNs) and a validation cohort (IPMNs diagnosed on imaging), demonstrated that blood-based extracellular vesicles (EVs) allow differentiating high-risk IPMN from low grade and noninvasive pancreatic cystic lesions [88]. Using a novel digital EV screening method technique, Yang et al. evaluated 22 plasma-based markers that were observed to be differentially expressed in pancreatic ductal adenocarcinoma (PDAC), especially mucin-based markers (MUC1, MUC2, MUC4, MUC5AC, MUC6, and MUC13), as well as molecules strictly related to PDAC vesicles (EpCAM, EpHA2, Glypican 1, STMN1, and TSP1) [89,90]. In IPNM, MUC5AC is expressed in all the histological subtypes [91]. Its presence in circulating EVs only in those patients with invasive IPNM suggests that EVs can serve as potential sources of minimally invasive biomarkers [78]. Although this finding lies on a limited number of patients, and most of them underwent surgical resection with histopathologic correlation, it suggests that EV profiling has the potentials to transform IPMN malignancy detection and surgical evaluation. A confirmation of the result within a larger cohort including non-operative candidates undergoing surveillance would thus have high clinical relevance.

### 3.4. Concept of Liquid Biopsy 

Blood-detectable genetic alterations associated with tumors can be clinically valuable as a non-invasive alternative to traditional biopsies, with application from early detection of disease recurrence to monitoring treatment response and the emergence of drug resistance [92]. Among these “liquid biopsies” that analyze circulating nucleic acids for cancer diagnosis, circulating cell-free tumor DNA (ctDNA) is probably the most clinically advanced approach [93]. Combined with circulating tumor cells (CTCs) analysis, it may represent a tool to assess in real-time both tumor burden and molecular features of the disease [94]. ctDNA has been studied in patients with various cancer types, including advanced pancreatic cancer [95,96]. A recent meta-analysis including 19 studies further evaluates ctDNA and other liquid biopsy diagnostics, such as CTSs and blood exosomes, in PDAC detection [97]. For the overall liquid biopsy, they found sensitivity, specificity, and area under the ROC curve were 0.80, 0.89, and 0.94, respectively. Among the different detection methods, exosomes showed the highest sensitivity and specificity, confirming the strong diagnostic value of liquid biopsy in detecting pancreatic cancer. The utility of liquid biopsy has been extended in cyst-type classification patients with pancreatic cystic neoplasms (PCN) [98]. In IPMN, the positive prevalence of GNAS mutations in circulating cfDNA was found significantly high (70%), even more in IPMN with intestinal subtypes, whereas KRAS mutations were nearly absent. GNAS mutation was detected also in cyst fluid and duodenal and pancreatic juice samples of patients with PCN [99,100]. Very recently, Hata et al. reported a pilot study taking into account GNAS alterations in ctDNA obtained from 57 patients with histologically diagnosed pancreatic cystic neoplasms (PCNs), 34 of which presenting IPMN [48]. They found that GNAS in ctDNA from peripheral blood of patients with pancreatic cysts was significantly higher in those with IPMN so this mutation can serve as a specific IPMN predictor for differentiating it from the various PCNs. In addition, the prevalence of GNAS was higher in IPMN with intestinal subtypes rather than the other subtypes. However, GNAS was not accurate enough in distinguishing the different histological grades of IPMN. Thus, ctDNA may not only serve as a biomarker of malignancy but may also be a useful method for non-invasive cyst classification. The use of DNA-based techniques appears a promising route to early detection of pancreatic cancer, although its role in detecting IPMN associated invasive cancer has to be more investigated [101].

## 4. Discussion and Conclusions

**Figure 2:**
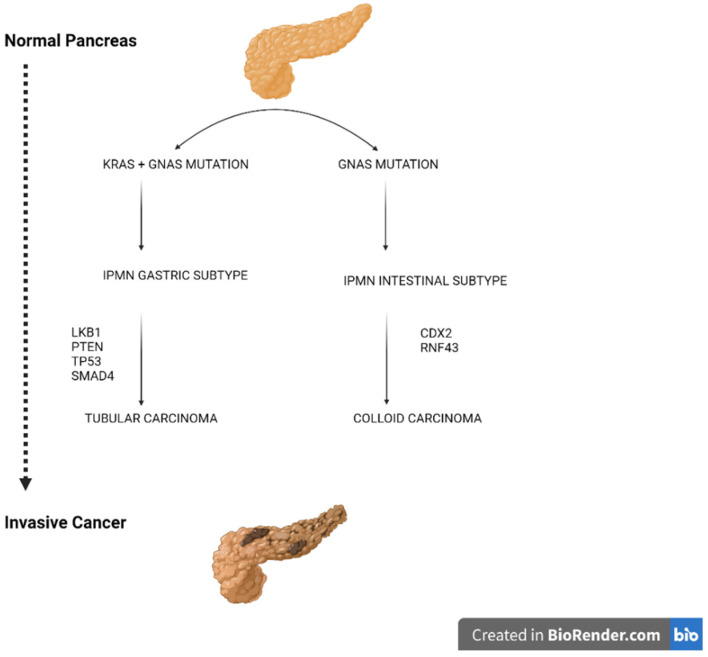
Hypothetic pathways of progression from the normal pancreas to intestinal or gastric IPMN and degeneration to pancreatic cancer.

Pancreatic cancer is a rising global burden in terms of morbidity and mortality. Pancreatic cancer precursors include PanIN (the most common), mucinous cystic neoplasm, and IPMN [102]. The progression from PanIN to invasive carcinoma has been widely investigated; however, the microscopic dimension of this lesion does not allow the detection with cross-sectional imaging representing a limit in cancer prevention [103]. Differently, pancreatic cyst lesions can be easily detected with cross-sectional imaging and are frequently found incidentally in 2.6% of the general population [104]. However, the molecular mechanism of progression from IPMN to invasive carcinoma is less well understood. Patra C. K. et al., in a murine model of IPMN related pancreatic cancer, demonstrated the cooperation of GNAS with KRAS and p53 in tumor initiation, progression, and malignancy maintenance [105]. Although pancreatic GNAS alteration is specifically associated with IPMN, its mutation alone is insufficient to induce the occurrence of IPMN requiring the concurrent presence of other genetic alterations (e.g., KRAS mutations and p53 loss of function) [106]. Other murine models showed the occurrence of IPMN with the association of KRAS mutations with other mutations (e.g., LKB1 or PTEN) without GNAS alterations [107]. The early mutation of KRAS and GNAS in association with CDX2 expression and RNF43 alterations has been correlated with the differentiation of IPMN toward the intestinal subtype. In this setting, GNAS mutations lead to carcinogenesis with the occurrence of colloid type malignancy. Differently, the association of KRAS mutations with PTEN or LKB1 alterations induces the differentiation toward the non-intestinal subtype of IPMNs leading to a tubular-type carcinoma [71]. The accumulation of other mutations such as p53, CDKN2A, and SMAD4 occurs in advanced lesions leading to cancer progression and maintenance (Figure 2) [71].

The risk of malignancy in IPMN varies depending on the subtype (SB-IPMN, MD-IPMN, or MT-IPMN), the histological pattern (gastric, intestinal, pancreatobiliary, and oncocytic), and the morphological presentation (e.g., presence of worrisome features). In a surgical series, the risk of malignancy in MD-IPMN and MT-IPMN ranged respectively from 6 to 46% and from 60 to 92% [108]. Conversely, the risk of malignancy in SB-IPMN is lower. Balduzzi A. et al. performed a meta-analysis of 24 studies including 8941 patients with SB-IPMN [108]. They reported that 20.2% of patients presented a progression during the follow-up with a pooled incidence of malignancy of 3.5% (range 0–32.8%); among patients undergoing surgical resection, 29.5% showed malignancy (including both high-grade dysplasia and invasive carcinoma) at the final histological analysis. The occurrence of concomitant PDAC was 0.8% (range 0–7%); 0.5% of patients showed distant metastasis during the surveillance. Histologically, IPMN can be distinguished into four subtypes: intestinal, gastric, pancreatobiliary, and oncocytic. The progression of the intestinal subtype leads to colloid carcinoma, while the other subtypes are associated with ductal adenocarcinoma [109]. From the main duct originate the intestinal, pancreatobiliary, and oncocytic subtypes meanwhile from the branch duct the gastric subtype [91]. The prognostic value of the histological subtypes is still today controversial as the available data are conflicting. The pancreatobiliary subtype is generally considered the most aggressive IPMN, thus it presents a stronger association with malignancy, a higher rate of recurrence, and poorer overall survival [110]. The presence of the high-risk stigmata and the worrisome features defined by the 2017 Fukuoka Consensus Guidelines increases the risk for malignancy and requires further investigations or surgical treatment [25] (Table 1).

Still today, the only curative approach to IPMN with suspicion of malignancy is surgery. Duodeno-cephalo-pancreatectomy, distal pancreatectomy, middle pancreatectomy, and total pancreatectomy are the available surgical techniques. However, pancreatectomy is considered one of the most challenging abdominal surgery and it is associated with high morbidity and mortality rate [111,112]. The indication for surgery should be given only for IPMN with high-grade dysplasia or invasive carcinoma. The best timing for surgery is before the occurrence of invasive carcinoma when the high-grade dysplasia is present. The detection of high-grade dysplasia and invasive carcinoma is often challenging, and cross-sectional imaging is frequently inadequate. For this reason, several humoral predictors of malignancy have been researched to stratify the risk of cancer occurrence and perform surgery in the best moment of the IPMN natural history (Table 2).

Classically, CA 19-9 and CEA are considered biomarkers of several malignancies including pancreatic cancer. High levels of CA19-9 (>37 U/mL) are associated with the presence of high-grade dysplasia and invasive carcinoma and can be used to distinguish between malignant from benignant IPMN with a specificity and a sensitivity of 89% and 40% respectively [35]. In addition, high levels of CEA (>5 µg/L) can predict the presence of malignant IPMN with a specificity of 96.4% and a sensitivity of 6.1% [23]. Both these tests present a high specificity but a low sensitivity with a consequent low negative predictive value. Moreover, the role of clinical scores such as NLR, PLR, and CAR have been evaluated as predictors of high-grade dysplasia and invasive carcinoma [39,45,50]. Although several studies have reported their utility in the prediction of malignant IPMN, a standardized cut-off is still today not available, and these scores are not widely used in daily practice. When the cross-sectional imaging and the seral biomarkers are inconclusive, a EUS examination with an FNA sample can be performed to research specific biomarkers directly in the cystic fluid. The cystic fluid sample can be used to perform a cytological analysis on one side to distinguish between mucinous and non-mucinous cysts with a sensitivity of 54% and a specificity of 93% [9]; on the other, it can be used to detect malignancy with a specificity of 83–99% and a sensitivity of 25–88% [59]. According to the Fukuoka guidelines, positive cytology for high-grade dysplasia or invasive carcinoma is an absolute criterion for surgical resection [58]. Moreover, the dosage of Mucins in the cystic fluid can be performed for the differential diagnosis between mucinous and non-mucinous cysts (sensitivity 80% and specificity 40%) and to detect degenerate IPMN (in degenerate IPMN an overexpression of MUC1, MUC2, and MUC4 and a down expression of MUC5A have been documented) [23,24,25]. Other chemical analyses can be performed on cystic fluid such as glucose, amylase, and CEA dosage. However, these markers can be used to distinguish between mucinous and non-mucinous cysts but are not useful to detect dysplasia or invasive carcinoma. Recently the cystic fluid DNA sequencing has been reported as a useful tool for the diagnosis of degenerate IPMN. Among the several genetic mutations, the detection of KRAS, GNAS, and RNF43 alterations is associated with malignant IPMN [36,75]. Moreover, the research of specific miRNA and telomeres alterations in the cystic fluid sample has the potential to diagnose invasive carcinoma [44,45,46]. More recently, the concept of liquid biopsy is gaining popularity. The research of cancer-associated genetic alterations in blood samples represents the new frontier of mini-invasive oncological diagnosis and monitoring [93,94]. The research of blood ctDNA and CTCs have been described for the diagnosis of several cancers. Moreover, EVs have been described for the early diagnosis of degenerated IPMN. However, the role of liquid biopsy in IPMN is still poorly understood and needs more investigation to be used in clinical practice [88].

In conclusion, many biomarkers have been studied in blood and cystic samples of patients with IPMN. These markers on one side can be used for the differential diagnosis between a mucinous and non-mucinous cyst, on the other side, few markers are available for the early diagnosis of high-grade dysplasia and invasive carcinoma. The cystic fluid DNA sequencing, the liquid biopsy, and the research of EVs present the potential of a mini-invasive diagnosis, but their role in daily practice is still under investigation. More studies are needed to find and validate new biomarkers to detect high-grade dysplasia and invasive carcinoma to perform a curative surgery at the best timing of IPMN natural history.

## Figures and Tables

**Table 1:** Worrisome features and high-risk stigmata for IPMN according to the 2017 Fukuoka Consensus Guidelines.

Worrisome Features	High-Risk Stigmata
Increased levels of CA 19.9 (>37 U/mL) Main pancreatic duct diameter 5–9.9 mm Cyst diameter >30 mm Enhancing mural nodules <5 mm IPMN-induced acute pancreatitis Thickened/enhancing cyst walls Cyst grow-rate >5 mm/2 year Abrupt change in caliber of the pancreatic duct with distal pancreatic atrophy Lymphadenopathy	Jaundice Enhancing mural nodule >5 mm Main pancreatic duct diameter >10 mm

CA 19.9 (Serum carbohydrate antigen 19-9), IPMN (Intraductal Papillary Mucinous Neoplasia).

**Table 2:** Humoral biomarkers predictors of degenerate IPMN.

Biomarkers	Description
Ca 19.9 (>37 U/mL)	89% sensitivity and 40% specificity in detecting degeneration.
CEA (>5 µg/L)	96.4% sensitivity and 6.1% specificity in detecting degeneration.
NLR (>2)	73.1% sensitivity and 58% specificity in detecting degeneration.
PLR	Not well-established cut-off. >200 associated in 83% to degeneration.
Cytological analysis	83–99% sensitivity and 25–88% specificity in detecting degeneration.
Cystic fluid mucins	Overexpression of MUC1, MUC2, and MUC4 and a down expression of MUC5A are associated with degeneration.
Cystic fluid DNA sequencing	The presence of KRAS, GNAS, and RNF43 is associated with degeneration.

## Data Availability

Not applicable.
